# Lysosomotropic Features and Autophagy Modulators among Medical Drugs: Evaluation of Their Role in Pathologies

**DOI:** 10.3390/molecules25215052

**Published:** 2020-10-30

**Authors:** Tatiana A. Korolenko, Thomas P. Johnston, Vaclav Vetvicka

**Affiliations:** 1 Federal State Budgetary Scientific Institution Scientific Research Institute of Physiology and Basic Medicine, Timakova Str. 4, 630117 Novosibirsk, Russia; t.a.korolenko@physiol.ru; 2 Division of Pharmacology and Pharmaceutical Sciences, School of Pharmacy, University of Missouri-Kansas City, Kansas City, MO 64108, USA; JohnstonT@umkc.edu; 3 Department of Pathology, University of Louisville, Louisville, KY 40292, USA

**Keywords:** lysosomotropic agent, lysosome, autophagy, autophagy modulators

## Abstract

The concept of lysosomotropic agents significantly changed numerous aspects of cellular biochemistry, biochemical pharmacology, and clinical medicine. In the present review, we focused on numerous low-molecular and high-molecular lipophilic basic compounds and on the role of lipophagy and autophagy in experimental and clinical medicine. Attention was primarily focused on the most promising agents acting as autophagy inducers, which offer a new window for treatment and/or prophylaxis of various diseases, including type 2 diabetes mellitus, Parkinson’s disease, and atherosclerosis. The present review summarizes current knowledge on the lysosomotropic features of medical drugs, as well as autophagy inducers, and their role in pathological processes.

## 1. Introduction

The concept of lysosomotropic agents (LA) was introduced by de Duve and coauthors soon after their discovery of a new class of subcellular organelles: lysosomes. This concept has had a great influence on the development of cellular biochemistry, biochemical pharmacology, and clinical medicine. According to this concept, LA include all drugs (of different chemical structures) that can be selectively concentrated inside lysosomes after in vivo administration and exert their effects on the cell via lysosomes [1], meaning that weak bases able to penetrate the membrane and accumulate inside lysosomes are considered to be LA. The acidic milieu of the lysosome results in the accumulation of weakly basic substances that can penetrate the membrane. Following entry into the lysosome, these molecules become charged, which inhibits reverse translocation out of the lysosome.

Lysosomes have acidic luminal pH (between 4.7 and 4.9), and hence, entrap a vast array of prognostic and therapeutic agents, all of which are hydrophobic, weakly-basic compounds: LA drugs [2]. Even though lysosomotropism has been extensively characterized and studied, the pathophysiological significance of this process is still not fully understood. It has been suggested that some LA hold promise for research into lysosomal functions and pathologies, especially intralysosomal storage syndrome, which can develop as an adverse side effect of various compounds. An additional potential side effect of these compounds is the prevention of mitochondrial membrane defects caused by the toxic action of chloroacetaldehyde. Since this molecule is not only a toxic metabolite in numerous industrial chemicals, but also occurs in drinking water, these findings might have significant clinical application [3].

Some LA drugs related to cell death were later shown to be promising for tumor treatment. Many drugs in clinical practice are characterized by special effects on the cell, including vacuolization, changes in lysosomal-membrane permeability, an increased number and size of lysosomes, and, in some cases, accumulation of undegraded material. These molecules have significant therapeutic activity and are used in clinical practice for treatment of several psychoses, malaria, and depression (for review, see Kuzu et al. [4]). New biological features of these agents, e.g., antiviral activity (chloroquine (CQ) compounds), and the ratio of beneficial to adverse (side) effects are described here. We discuss results of the use of several types of LA drugs in different fields of experimental biology and clinical medicine, including oncology, liver damage, atherosclerosis, and some inflammatory diseases. General information about these drugs is shown in Table 1.

Autophagy represents a cellular recycling system present in almost every type of cell. It is a catabolic process important for degradation of proteins and damaged subcellular structures; however, recently, autophagy has been found to be related to the catabolism of lipids [31]. The ability of autophagy to contribute to the maintenance of lipid metabolism is becoming particularly relevant for genetic lysosomal storage disorders where changes of autophagic flux have been suggested to contribute to the pathophysiology [31]. Triglycerides (TG) and cholesterol in lipid droplets (LD) can be taken up by autophagosomes and then delivered to lysosomes for degradation by acid hydrolases. Autophagy has attracted considerable attention as a target for the development of novel therapeutics [32,33]. Nevertheless, further research is necessary in this direction. On one hand, excessive autophagy may lead to autophagic death of macrophages, thereby further aggravating the inflammatory response [34]. On the other hand, administration of drugs improving the regulation of macrophage autophagy has become a novel treatment for sepsis. Autophagy modulators, especially autophagy inducers, hold promise [35], as a new strategy for the treatment of viral diseases [36]. Insulin activates intracellular transport of LD, triggers a release of TG from the liver [37]. Metabolic syndrome or obesity often causes abnormal lipid accumulation in LD in the liver, which is also called hepatic steatosis. New approaches to the treatment of metabolic syndrome and type 2 diabetes mellitus are possible because of new methods to activate lipophagy [38]. With exception of diabetes type 2, autophagy inducers hold promise for the prophylaxis and treatment of different diseases including Parkinson’s disease, atherosclerosis, fibrotic disease [39], and several types of cancer [40]. An additional important process is lipophagy, which upon its recognition, significantly altered our views of lipid metabolism [41]. The term lipophagy describes autophagic degradation of LD, and it has been demonstrated that lipophagy is crucial for the regulation of lipid stores [41]. Originally described in hepatocytes, it is the process that modulates lipid stores in fat storing cells. In general, it is a process connecting autophagy and lipid metabolism [42] and is often considered to be a new type of selective autophagy. The major role of lipophagy seems to be regulation of cholesterol efflux [43], and mediation of resistance to cell death [41]. The participation of macrophage lipophagy in reverse cholesterol transport has been discussed by Jeong et al. [44]. This mechanism mobilizes cholesterol from LD of macrophages via autophagy and lysosomes. Mechanisms associated with the cholesterol efflux pathway in macrophagic foam cells and the role of lipophagy as a therapeutic target hold promise for the prophylaxis and treatment of atherosclerosis and other disorders related to intracellular lipid storage (type 2 diabetes mellitus and some lysosomal storage diseases) [45,46] and some parasitoses [47]. In such cases, a natural question arises concerning the use of autophagy inducers [48].

As suggested above, lipophagy regulates intracellular lipid stores, cellular levels of free lipids and fatty acids, and energy homeostasis. Disturbances in lipophagy can cause excessive lipid accumulation in a tissue (e.g., in hepatic steatosis), may alter hypothalamic neuropeptide release to affect body weight, can block cellular transdifferentiation, and sensitize cells to death stimuli [41]. Mechanisms responsible for regulating lipophagy have strong impact on various physiological and pathological conditions [45].

## 2. Low-Molecular-Weight Weak Bases in Experimental and Clinical Medicine

Low-molecular-weight lysosomotropic lipophilic weak bases (e.g., CQ and its analogs and some antitumor drugs) [1], undergo marked sequestration and concentration within lysosomes, thereby changing lysosomal functions [49]. Additionally, studies have revealed increased sensitivity of cancer cells to certain LA and suggested that the actions of LA may potentially represent a promising approach for selective destruction of cancer cells. The entrapment of these drugs in lysosomes has been demonstrated to be luminal drug compartmentalization. According to recent data, LA weak bases modify pH-dependent membrane fluidization and undergo intercalation and concentration within the lysosomal membrane. The latter phenomenon has been proven experimentally (as increased free activity of lysosomal enzymes, such as acid phosphatases in lysosomes, in comparison with no-treatment controls), by morphological methods with the help of confocal microscopy involving fluorescent compounds, and by means of drugs delivered inside the lysosomal membrane, followed by molecular dynamics modeling of the pH-dependent membrane insertion and accumulation of various LA weak bases, including some anticancer drugs [49].

In addition, LA, such as l-leucyl-l-leucyl methyl ester (Leu-Leu-OMe), induce cell death via lysosomal membrane permeabilization of mast cells. As this action is rather selective for mast cells alone, this compound can be administered systemically [50]. For details of the impact of LA molecules on lysosomal membrane permeabilization, see Villamil Giraldo et al. [51].

### 2.1. Chloroquine

This drug has primarily been used to prevent and treat malaria in geographic areas where malaria remains sensitive to this treatment and in the treatment of other diseases like amebiasis (an antiparasitic effect), rheumatoid arthritis (an anti-inflammatory effect of this drug), and autoimmune disease (systemic lupus erythematosus). Following its introduction in clinical medicine, the lysosomotropism of CQ was reported. This compound is widely used as a specific inhibitor of intralysosomal proteolysis in isolated cells (such as hepatocytes and macrophages). According to Mauthe et al. [52], this drug inhibits autophagic flux by decreasing autophagosome-lysosome fusion. It was demonstrated in vitro that CQ reversibly inhibits purified cathepsins H (CH), B (CB), and L (CL) at concentrations less than those observed inside lysosomes in vivo [53]. Chloroquine administration did not induce any changes of carbon particle phagocytosis by liver macrophages, modifications of fluid-phase (^125^I-PVP) uptake, and receptor-mediated endocytosis (^125^I-asialo-fetuin uptake). However, instead of direct effects on phagocytosis, CQ and similar LA improve depression of macrophage function caused by FcR-mediated phagocytosis [54]. Chloroquine administered in vivo reproduces some symptoms of lysosomal storage diseases (especially during repetitive administration).

In general, CQ accumulation in the rat liver after single and repeated administration also reproduces lysosomal changes resembling some symptoms of lysosomal storage diseases. Repeated treatment of rats with CQ results in increased activity of hepatic lysosomal enzymes (acid phosphatase and β-galactosidase) [54].

### 2.2. Antitumor Drugs with Lysosomotropism

Cancer cells are well known to have elevated lysosomal function, which they subsequently use for higher proliferation and metabolism. This fact offers a new experimental approach to cancer therapy via lysosomal membrane permeabilization (for review, see Dielschenider et al. [55]).

It has been shown that TFEB (transcription factor EB; a protein encoded by the *TFEB* gene in humans) is activated after exposure of cancer cells to lysosomotropic anticancer drugs, thus, causing lysosome-mediated cancer drug resistance via increased lysosomal biogenesis, lysosomal drug sequestration, and drug extrusion through lysosomal exocytosis [56,57]. Zhitomirsky et al. [57] studied the molecular mechanism underlying LA-induced activation of TFEB. It was demonstrated that their accumulation leads to membrane fluidization of lysosome-like liposomes, and this effect is strictly dependent on the acidity of the liposomal lumen.

Pleiotropy in biological systems and targeting of such systems allows many pharmaceuticals to be employed for multiple therapeutic purposes. Due to the LA features of CQ, it was recently proposed for combined use (forming a less toxic complex) with other drugs (e.g., osteotide) as a new approach to the treatment of some tumors [58,59].

Liver cells, especially Kupffer cells (KC); which are merely liver macrophages), are known to accumulate LA [60]. In this article, we focused our studies that evaluate lysosomal changes in the liver (following administration of LA to experimental animals) and relate them to either toxic side effects or intended pharmacological action. Common features of lysosomal changes include the overload of liver lysosomes by indigestible material, an increased size and number of liver lysosomes, inhibition of several lysosomal enzymes, a secondary increase in the activity of some lysosomal enzymes, increased autophagy, and proinflammatory effect in macrophages. In addition, we also focused on the adverse effects of some LA drugs used in clinical and experimental medicine to date and discuss some of the characteristics of these drugs related to lysosomal storage disorders and lysosomal dysfunction.

Using a prostate cancer model, phosphoinositide 3 kinase (PI3K-AKT) inhibitor AZD5363 blocked autophagy but did not induce apoptosis. However, in combination with CQ, tumor volume was decreased by almost 85%, suggesting its potential use in cancer therapy [61].

As LA are known to inhibit, or at least reduce, autophagy, which is an important target in cancer treatment, they have been evaluated for use in cancer therapy. The potency of CQ in this regard is relatively low; therefore, more agents have been tested including mefloquine, ciprofloxacin, and levofloxacin. The most active was mefloquine, which produced significant anticancer activity in both hormone receptor–negative and –positive breast cancer cells [62].

A LA (sunitinib) modifies gene expression and induces incomplete autophagic processes resulting in activation of the NF-κB pathway and upregulation of CXCL5 [58]. Further experiments suggested that CXCL5 might serve as a biomarker of LA efficacy.

### 2.3. Autophagy/Lipophagy and Proteases

According to the concept proposed by de Duve et al. [1], lysosomotropism can be conferred artificially on almost any substance by suitable coupling with an appropriate carrier. Jacques [63] was one of the first authors to concentrate on the functions of lysosomes of reticuloendothelial cells and their role in endocytosis of LA compounds such as labeled horseradish peroxidase and insulin [64]. Many of the LA compounds used in vivo have manifested a common feature: the ability to induce autophagy. During the past two decades, accumulated evidence has revealed the intrinsic connection between macrophage function and autophagy. This research field is focused on the role of autophagy (both nonselective and selective types) in the regulation of inflammatory and phagocytotic functions of macrophages [65]. It has been shown that macrophages represent an important component of the innate immune system, and these cells are involved in the defense of other cells against invading pathogens, in the clearance of cellular debris, and in the regulation of inflammatory responses. Excessive autophagy may also lead to autophagic death of macrophages, which further aggravates the inflammatory response [34], as shown in sepsis.

During the last two decades, there has been increasing interest in research on the mechanisms associated with autophagy/lipophagy and their role in the pathogenesis of various diseases [66,67]. Many papers have been published, among them comprehensive reviews on autophagy by Levine and Kroemer [46,68], Parzych and Klionsky [69], and Saha et al. [70] and papers with a special focus on proteolysis, proteases, and autophagy [71,72]. It is worth mentioning the development of new approaches to clinical application of autophagy [32]. Growing evidence indicates that the involvement of lysosomal dysfunction in human diseases goes beyond rare inherited diseases, such as lysosomal storage disorders, and includes common neurodegenerative and metabolic diseases, as well as cancer [73]. Lipophagy-derived fatty acids undergo extracellular efflux via lysosomal exocytosis [74]. Inhibition of autophagy or of lysosomal lipid degradation may attenuate fatty-acid efflux, thus, highlighting crucial participation of lipophagy in the pathogenesis of fatty liver [74].

New data have been obtained about the function of macrophages in cholesterol metabolism and in reverse cholesterol transport and about the contribution of macrophage lipophagy to atherosclerosis, which is related to the newly discovered functions of the lysosomal apparatus of macrophages.

Recent studies have provided new insights into the mechanisms underlying lipophagy induction, as well as the consequences of this process for cell metabolism and signaling [75]. In lipid storage diseases (e.g., atherosclerosis), macrophages can be a major source of foam cells, which are a hallmark of atherosclerotic lesions. Consequently, the involvement of macrophage autophagy in the pathophysiology of atherosclerosis is being increasingly investigated [33].

Autophagy is a catabolic process through which defective or harmful cellular components are targeted for degradation via the lysosomal route. It can contribute to cellular lipid homeostasis, which is particularly important in genetic lysosomal storage disorders where changes of autophagic flux have been suggested to contribute to the etiology [31].

Autophagic degradation of LD, termed lipophagy, is a major mechanism that promotes lipid turnover in numerous cell types. LDs are dynamic and complex organelles regulating the storage and release of lipids to implement their fundamental functions in energy metabolism, membrane synthesis, and production of lipid-derived signaling molecules. The recent finding that LD can be selectively degraded by the lysosomal pathway of autophagy through lipophagy resulted in a new understanding of how lipid metabolism regulates cellular physiology and pathophysiology [76]. Many new functions of autophagic lipid metabolism have been identified in various diseases including liver disease, atherosclerosis, and type 2 diabetes mellitus.

Lipophagy was originally described in hepatocytes, where it plays a critical role in cellular energy homeostasis in obesity and metabolic syndrome. In vitro and in vivo studies have revealed selective uptake of LD by autophagosomes, and inhibition of autophagy has been shown to reduce β-oxidation of free fatty acids owing to the increased accumulation of lipids and LD. An important issue concerning this process that needs to be addressed is the possible cooperativity between lipases and autophagy-related proteins in the regulation of lipolysis; liver-specific deletion of the autophagy gene Atg7 increases hepatic fat content [77]. On the other hand, excessive lipophagy or stimulation of autophagy can be used for the prevention and treatment of liver diseases related to lipid storage (hepatic steatosis, steatohepatitis, and liver cirrhosis) [66].

Recent studies offer original and novel approaches to the investigation of mechanisms underlying lipophagy induction, as well as the consequences of this process for cellular metabolism and signaling [65,75]. Importantly, lipophagy can be directly or indirectly regulated by genes, enzymes, transcriptional regulators, and other factors [78]. Cytosolic LD have been shown to be present in most cell types [79]. Lipid droplets consist of a core comprised of neutral lipids (mainly TG and sterol esters) surrounded by a monolayer of phospholipids [80]. Lipid droplets vary in their structure, chemical composition, and tissue distribution; specifically, they are coated by several proteins (perilipin and other structural proteins), lipogenic enzymes, lipases, and membrane-trafficking proteins [80]. Lipid droplets have been identified in most organisms, where they serve to store energy in the form of neutral lipids. They are formed at the endoplasmic reticulum membrane, where neutral-lipid-synthesizing enzymes are located. Recent findings indicate that LD remain functionally connected to the endoplasmic reticulum membrane [81].

Alterations in macroautophagy (typical autophagy) are a common feature of lysosomal storage disorders and can play a major role in the pathogenesis of these diseases. Autophagy implements the degradation and recycling of proteins, lipids, carbohydrates, and organelles. Multiple defects in autophagy contribute to a lysosomal storage disorder called Niemann-Pick type C (NPC). The multiple defects include increased formation of autophagosomes, slowed turnover of autophagosomes secondary to impaired lysosomal proteolysis, and the delivery of stored lipids to lysosomes via autophagy [82]. This disorder is a lysosomal storage disorder characterized by progressive neurodegeneration and liver damage with neurovisceral atypical lysosomal lipid storage [83]. This neurodegenerative lysosomal disorder results in the storage of cholesterol and other lipids due to defects in either NPC1 (a transmembrane protein involved in cholesterol export from the lysosome) or NPC2 (an intralysosomal cholesterol transport protein) [84]. Deficiency of either protein leads to the accumulation of cholesterol and glycosphingolipids in late endosomes and early lysosomes [85]. The Npc1 mouse model has been studied at six ages spanning the pathological progression of NPC. Altered gene expression was identified at all ages, including changes in asymptomatic 1-week-old mice. Biological pathways featuring early alteration of gene expression included lipid metabolism, the cytochrome P450 enzymes involved in arachidonic acid and drug metabolism, inflammation, immune responses, mitogen-activated protein kinase and G protein signaling, cell cycle regulation, cell adhesion, and cytoskeleton remodeling. In contrast, apoptosis and oxidative stress appeared to be late pathological processes. Further analysis identified two secreted proteins with increased serum levels in NPC1 patients: galectin-3 (LGALS3; a proinflammatory protein) and CD.

Protein catabolism in macrophages, which is accomplished mainly through autophagic-lysosomal degradation, includes the ubiquitin-proteasome system and calpains as has been demonstrated in atherosclerotic vessels [68,86]. Macrophages are key mediators of vascular inflammation and regulate arterial remodeling by secreting inflammatory cytokines and proteases such as cysteine proteases: CB, CL, CH, and CS). Cathepsins are lysosomal peptidases involved in intracellular protein catabolism and in various other physiological and pathological processes, and these enzymes participate in innate and adaptive immunity and in the regulation of antigen presentation [87]. By utilizing a model of atherosclerosis in which macrophages are overloaded with lipids (i.e., foam cells), it was shown that CB, CL, and CS are overexpressed in advanced arterial lesions and have abnormal activities when compared to non-inflamed sites [72]. These authors demonstrated that the activity of CB, CL, and CS is suppressed by oxidized lipids and that the cysteine protease CS has a substantial function in the autophagic-lysosomal degradation pathway. Consequently, loss of cysteine cathepsin function results in autophagy derangement. Shotgun proteomics has confirmed autophagy dysfunction and revealed a pivotal role of CL in a putative cathepsin degradation network. Additionally, CB was reported to regulate the noncanonical NLRP3 inflammasome pathway by modulating activation of caspase 11 in KC [88].

With the assistance of light microscopy and immunoenzyme techniques, it has also been found that KCs isolated from Wistar rats and CBA mice stain for aspartate protease cathepsin D (CD), and that a significantly lower number of CD molecules are present in hepatocytes. It should be mentioned that weak staining was also noted in sinusoidal endothelial cells. In parenchymal cells, many of the positive granules were located around bile canaliculi. Latex phagocytosis increased the activity of CD in all KCs [89]. Moreover, KCs manifest functional heterogeneity that is related to their position in the liver acinus; specifically, KCs with a high endocytic activity, large and heterogeneous lysosomes, and high lysosomal enzymatic activities have been found in the periportal zone. The activity of CD, just as that of other marker lysosomal enzymes (e.g., acid phosphatase and arylsulfatase B), increases with age in KCs of the rat liver [90]. The heightened enzymatic activity in Kupffer and endothelial cells from the rat liver may reflect more efficient phagocytic capacity in these animals. CD content, expressed in micrograms of the enzyme per milligram of protein, is approximately 5- to 24-fold higher in KCs than in parenchymal cells [91]. CD expression is weaker in hepatocytes, but stronger in KCs, which increase in number and size and have more diverse lysosome-related profiles [92]. In an age-related study, it was shown that resveratrol (a natural product derived from the skin of grapes that delays age-related changes in the liver) may activate mitochondria (thereby, resulting in utilization of lipids) and may also activate KCs. In this way, a beneficial biological milieu may be created for hepatocytes. Both processes might be associated with improved liver function, which is clinically significant, since this organ is central to metabolic regulation [92].

Two types of autophagy are recognized: “nonselective” and “selective” degradation. Nonselective or “bulk” degradation is frequently induced by nutrient depletion. Selective degradation involves sequestering specific cellular components under nutrient-replete conditions. These authors reported that cathepsins attenuate macrophagic inflammatory activity under hyperlipidemic stress by regulating lysosomal function and mitochondrial quality control.

### 2.4. Lysosomotropic Gadolinium Compounds and Suppression of Liver Macrophages

Metals, namely lanthanides and transuranium elements, were included by de Duve et al. [1] as LA. Gadolinium (belonging to lanthanides) and plutonium are included in this group of metals. In fact, gadolinium compounds are used in clinical medicine as contrast agents in magnetic resonance imaging and magnetic resonance angiography procedures. In experimental medicine, gadolinium chloride (GdCl_3_) has been used to intentionally suppress KC [93,94].

Suppression of proinflammatory activity by the M1 population of KCs is a protective factor in certain KC-mediated diseases, but not in all toxic pathological disease states [95], such as in several types of liver injury [96], liver steatosis [97], myocardial ischemia [98], and tumor development [99]. The mechanism for this protective effect, as well as the influence of KC suppression on serum lipids, remains unclear [100]. However, liver steatosis is related to the development of atherosclerosis and lipidosis. The majority of authors attribute this effect to KC ablation (knockout) and suppression of their secretion of proinflammatory cytokines (IL-1), which may be important, but it should be noted that this is a very nonspecific, general reaction observed in many pathological disease processes.

Previously, autophagy has had an unknown function in regulating intracellular lipid stores (macrolipophagy) [101]. It has been suggested that LD and autophagic components are associated with nutrient deprivation, and that inhibition of autophagy in cultured hepatocytes and mouse liver increases TG storage in LD. However, it may be that decreased endocytosis by KC is regulated by autophagy during liver lipidosis, which occurs simultaneously with autophagy of cellular organelles [33,102].

#### Effect of Macrophage Suppression on Serum Lipids in Hyperlipidemic Mice

**Figure 1:**
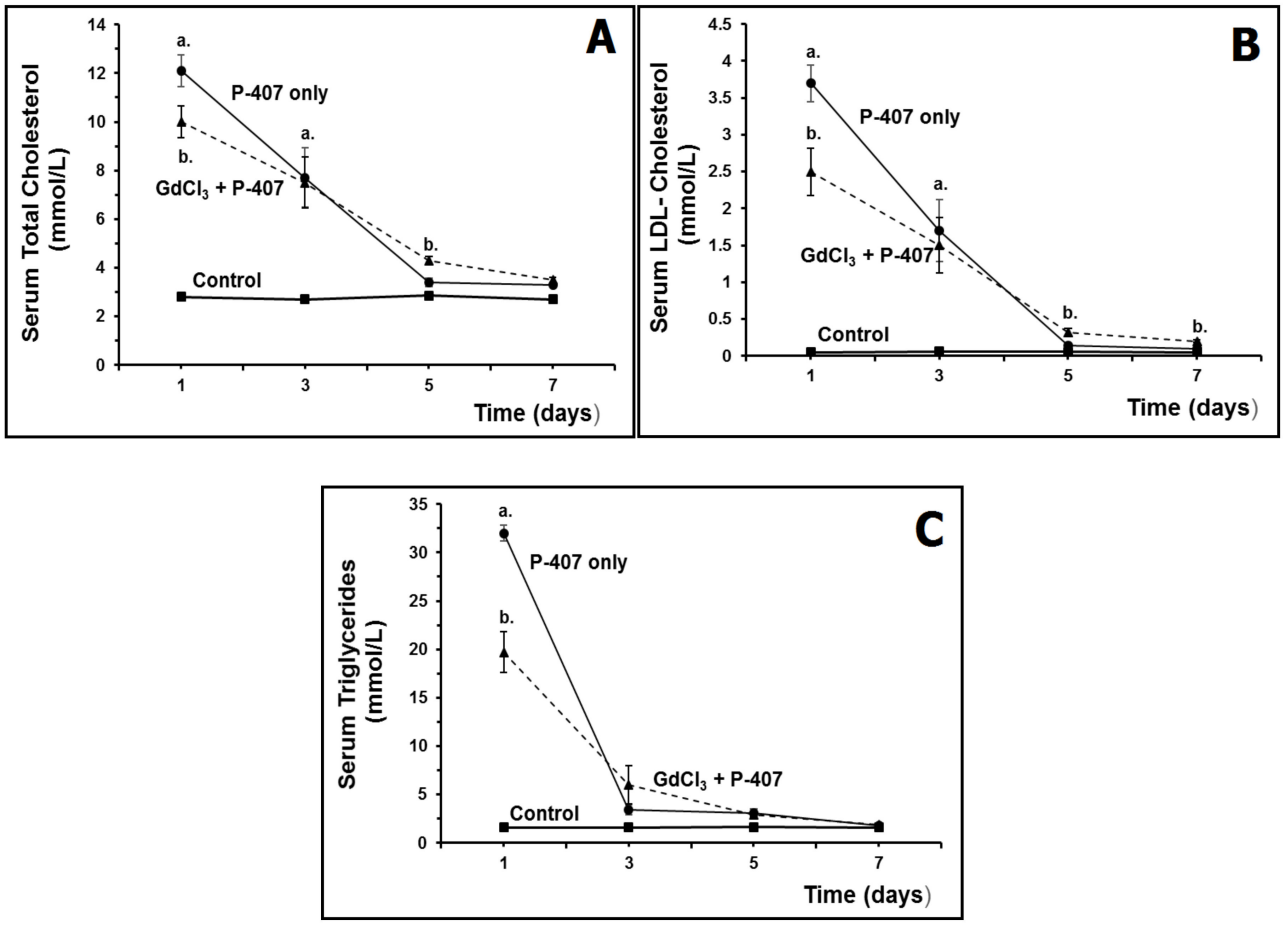
Effect of GdCl_3_-mediated macrophage suppression in P-407-induced hyperlipidemic mice. Data are the mean values ± standard error of the mean (SEM) of serum total cholesterol (**A**), LDL-C (**B**), and triglycerides (**C**) for P-407-induced lipemic mice (●), P-407-induced lipemic mice pretreated with GdCl_3_ (GdCl_3_ + P-407) (▲), and saline-treated control mice (■). The number of mice in each group was 9–12. Dashed line utilized to easily identify those mice receiving GdCl_3_. ^a^ *p* < 0.001 vs. control. ^b^ *p* < 0.01 vs. group dosed with P-407 only.

A mouse model of hyperlipidemia (developed by T.P. Johnston) induced by the administration of the block copolymer known as poloxamer 407 (P-407) has been widely used in experimental biology and medicine, see Johnston et al. for a comprehensive review of this model [103]. Using this well-documented mouse model of hyperlipidemia, it has been previously established that a single dose (300 mg/kg) of P-407 induces a significant increase in serum total cholesterol, LDL-C, and especially TG, at 24 h post-dosing when compared to saline-treated controls (Figure 1A–C), and that the hyperlipidemic state persists for approximately 5 days post-dosing, although TG returns to baseline (control) levels by three days following P-407 treatment (Figure 1C).

Using GdCl_3_ to intentionally suppress the function of KC in P-407-induced hyperlipidemic mice, we have evaluated whether diminished KC function affects the atherogenic serum lipid profile and the activity of proteases in the liver. KC suppression can be easily induced in mice using a single injection of GdCl_3_ at a dose of 10 mg/kg. Importantly, and, as an aside, there is a rapid uptake of gadolinium by the liver following a single intravenous dose of GdCl_3_ to mice. Specifically, we observed that 30% of an administered dose of GdCl_3_ was sequestered in the liver 5 min post-injection, reaching a maximum (~80%) at 1 h, and then decreasing to approximately 15% by day 5.

In our pilot study, mice were sacrificed after 1 day (i.e., the time point of maximum KC suppression at the expense of apoptosis and reduced endocytosis), and after 3, 5, and 7 days (i.e., the time point for ‘repopulation’ of KC). As mentioned above, P-407-treated mice served as the model of hyperlipidemia for this preliminary experiment.

In the preliminary study to determine how GdCl_3_-mediated suppression of KC affected lipid levels in P-407-induced hyperlipidemic mice, we observed that serum total cholesterol, LDL-C, and TG were significantly decreased at day 1 (i.e., time point for maximum macrophage depression) when compared to mice treated with P-407 only (Figure 1A–C). Three days after GdCl_3_ administration (i.e., KC suppression), serum lipids were similar in treated (P-407 + GdCl_3_) and untreated (P-407 only; no GdCl_3_) groups (Figure 1A–C). However, interestingly, we observed that serum total and LDL-C at day 5 were significantly increased in GdCl_3_ + P-407-treated mice when compared to mice treated with P-407 only (i.e., time point at which there is increased macrophage repopulation/restoration) (Figure 1A,B).

The mechanism(s) responsible for the effects of macrophage suppression on hyperlipidemia in mice have not been previously elucidated. However, based on our preliminary findings, we would suggest that they are primarily related to suppression of KC. Based on these preliminary results, KC overloaded with gadolinium (sequestered inside the lysosomes) were still able to incorporate additional lipids, which was verified in our electron microscopy analyses (i.e., the accumulation of LD in KC and the formation of autophagosomes). However, our preliminary findings may possibly suggest another mechanism for the reduction of serum lipids when KC populations are intentionally suppressed in mice with GdCl_3_; namely, other pools of macrophages (e.g., in the lung and spleen) assist with the removal of serum lipids as a compensatory mechanism when the function of KC are reduced. Interestingly, the numerical density of KC increases in P-407-induced hyperlipidemic mice and returns to baseline after 5 days in accordance with the cessation of hyperlipidemia.

It is interesting to note that P-407 is an effective general lipase inhibitor, and that it has been suggested that lipophagy, which is induced in P-407-treated mice, is merely autophagy-mediated degradation of LD by lysosomal acid lipase. Since P-407 inhibits lysosomal acid lipase in KC, the enzyme cannot catalyze the initial step of lipolysis of TG, which, as mentioned above, is an important mechanism for supplying KC with substrates used for energy [79]. Accordingly, autophagy is typically activated in lipase-deficient Mph to restore the ‘energy deficit’ created by defective lipolysis [79]. Thus, our preliminary studies to date would suggest that the effects of GdCl_3_ in P-407-induced hyperlipidemic mice may prove beneficial in more fully elucidating the role of autophagy in the regulation of lipid degradation/metabolism [102].

With regard to GdCl_3_-mediated KC suppression and the activity of hepatic proteases, we have also shown in preliminary studies (unpublished findings) that GdCl_3_-treated mice show a significant increase in the activity of the aspartate protease, CD, for up to 3 days following administration of GdCl_3_. Moreover, we determined that the activity of CB in hepatocytes does not change after GdCl_3_ administration, although the activity of cathepsin L is significantly reduced. Finally, it is worth mentioning that the activity of cathepsins are not only affected by GdCl_3_, but also P-407, since our preliminary experiments (unpublished findings) demonstrated that the administration of P-407 alone to mice results in a significant increase in the specific activity of CB and CL in liver tissue relative to controls.

## 3. High-Molecular-Weight Lysosomotropic Agents

The lysosomal system plays the main role in intracellular catabolism of naturally occurring endogenous and exogenous macromolecules and subsequent recycling of their monomeric components [104,105]. Drugs with lysosomotropic features used in clinical practice are characterized by specific effects, including cytoplasmic vacuolization, an increase in the number and size of lysosomes, inhibition of their enzymes, and accumulation of undegraded material, leading mainly to phospholipidosis [2]. With the help of LA, it is possible to set up pharmacological models reproducing intralysosomal storage syndromes. Some LA drugs can reach very high concentrations in cells and tissues, resulting in a very high volume of distribution, very low plasma concentrations, and often-extended half-times/half-lives in tissues [1,106]. These characteristics have been documented for some high molecular weight polymers used in experimental medicine to create models of lysosomal storage diseases and their treatment. A special place among such drugs belongs to Triton WR 1339, which has played an important part in the progress in the research on lysosomal functions in physiological and pathological conditions.

Nonionic detergent Triton WR 1339, a viscous polymer of the alkyl aryl polyether alcohol type, is classified as a nonionic surfactant, and has been important for the discovery of lysosomes, study of lysosomal functions, and the development of new methods of cellular biochemistry (isolation of overloaded lysosomes by the methods of isopycnic centrifugation, centrifugation in a density gradient of metrizamide, and others). Triton WR 1339 is taken up selectively by lysosomes (primarily by liver cells); thus, by changing their density, it has been possible to separate so-called Triton-filled lysosomes (tritosomes) using new centrifugation methods [107,108].

**Scheme 1:**
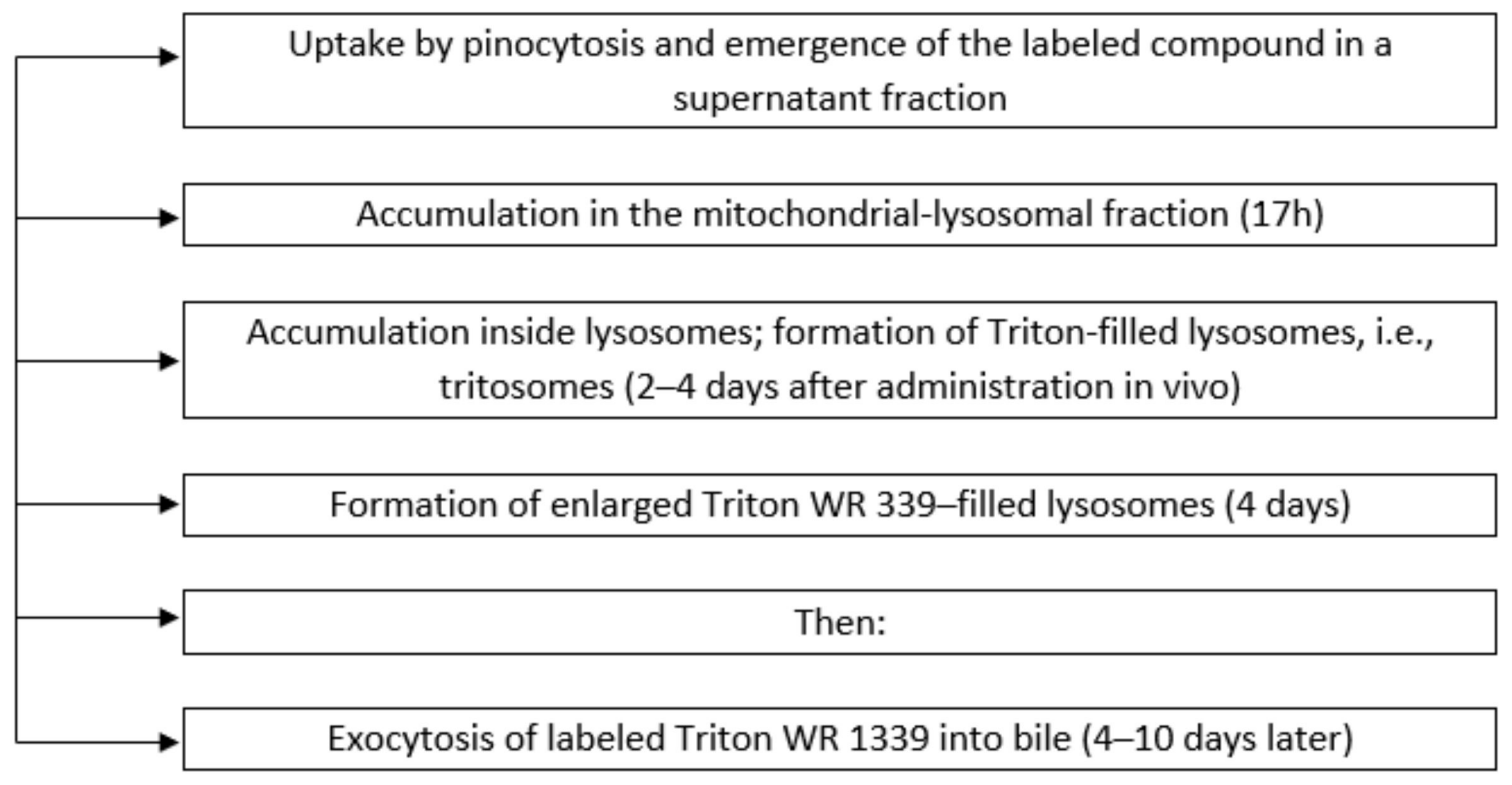
Intracellular “traffic” of ^131^I-labeled Triton WR 1339 in rat hepatocytes after intraperitoneal administration of this compound.

Wattiaux et al. [108] presented results that prove lysosomal localization of labeled Triton WR 1339 injected in vivo. Using methods of subcellular fractionation of rat liver homogenates in combination with centrifugation in a density gradient (Scheme 1), these authors demonstrated a similarity of distributions of radioactively labeled Triton WR 1339 and markers of lysosomal enzymes—acid phosphatase and other marker lysosomal enzymes—colocalized in the same peak [108]. It was shown that, similar to some labeled proteins (e.g., insulin), Triton WR 1339 first appeared in a supernatant fraction (slow uptake by fluid-phase pinocytosis), then “relocated” to the mitochondrial-lysosomal fraction, and ultimately reached the lysosomal peak on the 4th day after administration. The decreased density of the isolated tritosomes was a result of increased content of light lipids in these organelles, as well as the filling of tritosomes by light Triton WR 1339 [109]. The consequences of Triton WR 1339 accumulation were a significant increase in the number and size of lysosomes and changes in the inner structure of these organelles.

Due to the isolation of purified tritosomes, studies have been continued regarding the chemical composition of lysosomes (tritosomes) and lysosomal functions. At the same time, injection of Triton WR 1339 into animals (rats and mice) has become a new pharmacological model of lysosomal storage diseases, holding promise for investigations aimed at elucidating the pathogenesis of lysosomal storage syndrome and its treatment and prophylaxis.

### 3.1. Characteristics of the Membranes of Liver Lysosomes of Animals with Intralysosomal Storage Syndrome Induced by Triton WR 1339

Triton WR 1339 is rapidly taken up by the liver, where it accumulates in lysosomes and causes autophagic-vacuole formation [110]. Since it is excreted into bile, it may possibly explain the reduction in biliary phospholipid and cholesterol output. According to our data obtained on mice after a single administration of Triton WR 1339 (500 mg/kg, 4 days after), this agent causes labilization of lysosomes (an increase in the free activity of acid phosphatase, as compared to control mice receiving physiological saline) and increases the susceptibility to lysis by osmotic treatment in vitro in a hypotonic medium (i.e., a 0.125 M sucrose solution) [5]. These changes were a result of an increased volume of tritosomes, as evidenced by electron microscopy of hepatocytes and liver macrophages isolated from the experimental mice. Nonetheless, the regulatory mechanism of lysosomal stability is poorly understood to date. As has been mentioned earlier in this review, lipophilic or amphiphilic compounds with a basic moiety become protonated and trapped within lysosomes, and such lysosomotropic behavior is also documented for many pharmaceutical drugs [51]. The consequences associated with overloading lysosomes with LA has been discussed in several reviews, with a focus on the adaptation of lysosomes to these drugs [111].

### 3.2. Characteristics of the Hyperlipidemia Induced by Triton WR 1339

Triton WR-1339 (Tyloxapol) has been used to induce hyperlipidemia in animals, which develops possibly because of inhibition of lysosomal lipoprotein lipase. The low-density lysosomes (d = 1.00 to 1.13) from Triton WR-1339-treated rats have approximately 4- and 3-fold higher contents of TG and cholesterol, respectively, than do high-density lysosomes (d > 1.30) from silver colloid-treated rats [109]. This finding has been the basis for new studies involving the Triton WR 1339-induced model of hyperlipidemia.

The liver is the major organ responsible for cholesterol transport, metabolism, and excretion. The hyperlipidemia induced by Triton WR 1339 is characterized by elevated serum concentrations of total cholesterol, LDL-C, and very low-density lipoprotein cholesterol (VLDL-C), as well as decreased high-density lipoprotein cholesterol levels [112].

The hepatic production rate of TG is typically measured by temporal quantitation of increases in plasma TG concentration under conditions where TG hydrolysis by lysosomal lipoprotein lipase is inhibited [110]. The nonionic detergent Triton WR-1339 has commonly been employed to inhibit lipoprotein lipase. Another nonionic detergent mentioned earlier; specifically, P-407, also inhibits the activity of lipoprotein lipase. It has been demonstrated that P-407 is comparable to Triton WR 1339 in terms of the measurement of hepatic TG production, but is devoid of the unwanted effects of Triton [110,113]. Triton WR 1339, in addition to inhibiting TG hydrolysis, has physicochemical properties that may adversely affect lipoprotein metabolism.

Hayashi et al. [109] proposed that changes in the density of tritosomes are a consequence of intralysosomal storage of not only light Triton WR 1339, but also some lipids (LPs). Considering these results and the fact that the density of Triton WR-1339 is quite high (d ≥ 1.20), the decrease in the density of hepatic lysosomes upon Triton WR-1339 administration cannot be simply due to incorporation of the detergent, but rather may be a result of incorporation and accumulation of some lipid(s) (possibly, LP) in lysosomes together with Triton WR-1339.

There is a now an emerging trend regarding research on lysosomal membranes; namely, studies on the stability and permeability of lysosomal membranes in relation to the effects of so-called stabilizers and labilizators of lysosomal membranes [5,51]. In general, the overloading of lysosomes in vivo with different compounds induces labilization of lysosomes (exacerbates pathology) and increases osmotic pressure of lysosomes, but the role that these changes have on lysosome stability is still debated. Permeabilization of the lysosomal membrane and a release of hydrolytic enzymes into the cytosol accompanies apoptosis signaling in several systems. In some cases, increased permeabilization of the lysosomal membrane is followed by cell damage, and this approach may be used in cancer therapy [114].

**Figure 2:**
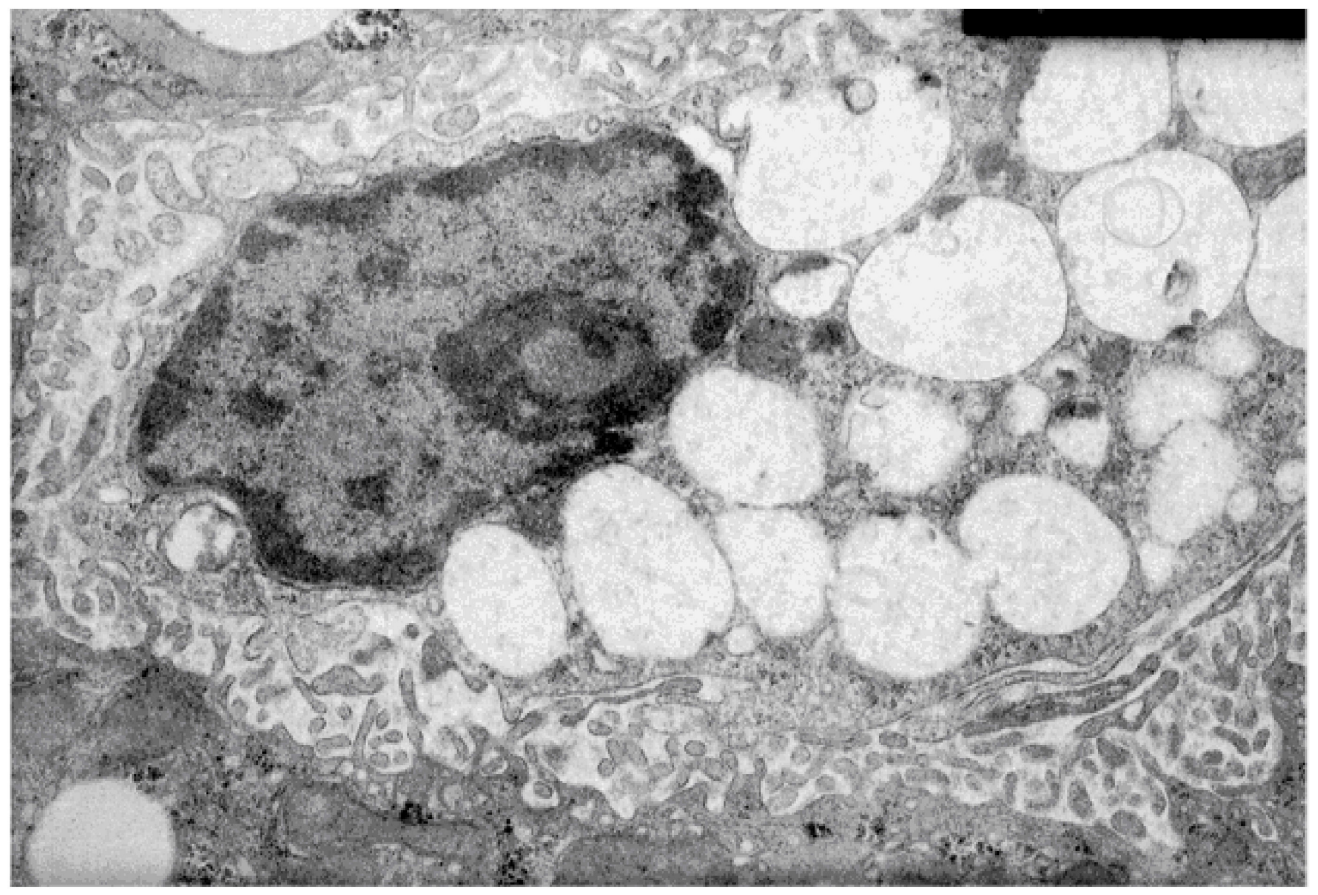
An electron micrograph of a murine liver macrophage after single intraperitoneal administration of lysosomotropic agent (LA) Triton WR 1339 (500 mg/kg, 4 days after); droplets containing lipids are seen in the cytoplasm.

In vivo, Triton WR 1339 influences liver macrophages, forming numerous so-called triton-filled lysosomes (Figure 2). However, in vitro, Triton WR 1339 exposure manifests dose- and time-dependent cytotoxicity. Triton WR 1339 treatment damages RAW 264.7 cells more than NIH/3T3 cells. All of the cells exposed to Triton WR 1339 show some morphological features of apoptosis, such as chromatin condensation and cell shrinkage [115].

In an experiment involving repeated administration of Triton WR 1339 to mice, the only resultant morphological change was the emergence of lipids and acid phosphatase (marker enzyme of lysosomes) in both hepatocytes and KCs; following discontinuation of Triton WR 1339 treatment these changes virtually disappeared in hepatocytes, however, they became more accentuated in KCs [116].

### 3.3. Autophagy Induction

Autophagy plays an important role in normal cell physiology and in the pathophysiology of several diseases. Dual functions of autophagy in tumor biology have also been demonstrated. For example, autophagy activation can promote cancer cell survival (protective autophagy) or contribute to cancer cell death (cytotoxic/non-protective autophagy) [117].

Compounds that induce autophagy may have broad therapeutic applications, including Tat-beclin 1 (derived from a fragment of an autophagy protein, beclin 1, which binds human immunodeficiency virus (HIV)-1 Nef), which is a potent inducer of autophagy and interacts with a newly identified negative regulator of autophagy, GAPR-1 [118].

Inhibitors of autophagosome/autophagolysosome formation include 3-methyladenine (an endosomal acidification inhibitor and autophagy inhibitor); bafilomycin A1 (an endosomal acidification inhibitor and autophagy inhibitor); chloroquine (a LA agent, inhibitor of endosomal acidification, and an autophagy inhibitor), and wortmannin (a PI3K inhibitor and autophagy inhibitor).

Metformin and trehalose (TH) are also in the family of autophagy inducers. Metformin is well known in medicine in terms of the treatment and prophylaxis of metabolic syndrome and type 2 diabetes mellitus. Obesity followed by metabolic syndrome and type 2 diabetes mellitus [119] is characterized by increased LD in the liver (and in microglia), because of steatosis and steatohepatitis. Recently, with the help of single-cell technologies, researchers revealed significant heterogeneity within the macrophage pool in both the liver and adipose tissue in obesity. LD-accumulating microglia represent a dysfunctional and proinflammatory state in the aging brain [120].

TFEB has been shown to increase lysosomal lipid catabolism and lipolysis by regulating lysosomal biogenesis [33]. TH (digested by enzyme trehalase), as well as trehalase-indigestible analogs (lactulose and melibiose), have therapeutic effects on neurodegeneration [121].

Fan et al. [122] identified several novel autophagy-inducing phytochemicals including Rg2 (a steroid glycoside chemical) activating autophagy in an AMPK-ULK1-dependent (AMPK activates autophagy by phosphorylating ULK1) and mTOR-independent manner. Induction of autophagy by Rg2 enhances the clearance of protein aggregates in a cell-based model, improving cognitive behaviors in a mouse model of Alzheimer disease, and prevents high-fat diet-induced insulin resistance.

Autophagy induction has been documented previously in cases of poisoning by heavy metals like cadmium and arsenic compounds [123]; ingestion of small doses of these compounds (in relation to ecological problems) was also found to be dangerous. Induction of autophagy has likewise been documented in experiments with gadolinium-based contrast agents [124]. Additionally, it should be noted that exercise (increased physical activity) can regulate LD dynamics in the normal and fatty liver by improving lipid metabolism [125].

TH is a non-reducing disaccharide with two glucose molecules linked through an α,α-1,1-glucosidic bond [126]. In fact, it has received substantial attention in the past few decades due to its participation in neuroprotection, especially in animal models of various neurodegenerative diseases such as Parkinson and Huntington diseases. TH, a modifier of the autophagy-lysosome system in macrophages, is a naturally occurring disaccharide with autophagy-inducing properties and has been shown to reduce atherosclerosis and atherosclerotic-plaque complexity. It has been reported that an uncharacterized transcriptional component of TH-dependent autophagy-lysosome induction in macrophages is associated with increased levels and nuclear translocation of TFEB, which is an activator of a broad network of autophagic and lysosomal genes [16]. In general, those authors have characterized the disaccharide TH as a novel inducer of TFEB with similar atheroprotective effects. Like TFEB overexpression, TH was able to reduce polyubiquitinated protein aggregates, IL1B levels, and apoptosis in cultured macrophages. In vivo, TH reduces atherosclerosis and plaque complexity in macrophages [16,33]. There are also data indicating that TH is a potent blocker of the autophagic flux [18]; however, this hypothesis requires further investigations.

In our experiments, a positive effect of TH (drinking of a 2% solution for 15–30 days) on the development of metabolic syndrome was noted in *db/db* mice (2 and 3 months old). Male and female diabetic (*db/db*) mice manifest severe obesity and hyperglycemia (in contrast to untreated CBA mice) and have a significantly reduced brain weight. TH treatment for 15 or 30 days significantly decreased blood glucose levels in both age groups of treated *db/db* mice as compared to untreated *db/db* mice and there was improvement in cognitive behavior. The diabetic (*db/db*) mice of both ages had an increased relative number of polymorphonuclear cells (*p* < 0.01) in peripheral blood, indicating an inflammatory reaction. However, TH treatment decreased the polymorphonuclear cell number in 2-month-old *db/db* female mice (*p* < 0.05), which would suggest some degree of anti-inflammatory action.

### 3.4. Autophagy Regulation

Dysregulation of autophagy is a relatively common feature in lysosomal storage diseases [127]. The serine/threonine protein kinase (TOR kinase), represents key target for autophagy regulation [128]. It has catalytic component formed by two complexes: mTORC1 and mTORC2. The former complex is sensitive to rapamycin, and is involved in various biological functions, such as cell growth, metabolism, and nutrient regulation, while the latter one is completely resistant to rapamycin and regulates functions, such as actin cytoskeletal organization, protein kinase Cα, and protein kinase B (Akt) kinase activity.

The 5′-adenosine monophosphate-activated protein kinase (AMPK), which is known as an energy receptor, activates autophagy by inhibiting mTORC1 and phosphorylation of raptor under glucose starvation. AMPK-mediated autophagy induction can also directly induce phosphorylation of ULK1, VPS34, and beclin-1 independently of mTOR.

In the brain, this mechanism has mostly been investigated in neurons, where the delivery of toxic molecules and organelles to lysosomes by autophagy is crucial for neuronal health and survival. Nevertheless, it was proposed that the dysregulation of autophagy in microglia also affects innate immune functions such as phagocytosis and inflammation, which, in turn, contribute to the pathophysiology of aging and neurodegenerative diseases [129]. In neurons, the autophagic-lysosomal pathway plays an important role in the clearance of protein aggregates caused by ischemia-induced neuronal endoplasmic-reticulum stress [130]. It has been suggested that moderate activation of autophagy may prevent neuronal damage to a certain extent, whereas excessive autophagy may be a contributing factor in neuronal death in cerebral ischemia and hypoxia. The dual role of autophagy has still not been completely elucidated.

Both brain and blood macrophages (known as microglia and monocytes, respectively) are a topic of interest for investigators trying to find new approaches to the treatment of neurodegenerative diseases [131]. Microglia become progressively activated and dysfunctional with age, and genetic studies have linked these cells to the pathogenesis of a growing number of neurodegenerative diseases [120]. Among microglial cells, “LD-accumulating microglia” are defective as it pertains to phagocytosis, produce large amounts of reactive oxygen species, and secrete proinflammatory cytokines. High-throughput RNA sequencing analysis of these cells has revealed a transcriptional profile (shaped by innate inflammation) that is distinct from previously reported microglial states. These authors described the basic concepts of autophagy and its regulation, discussed key points regarding its accurate monitoring at the experimental level, and summarized the evidence linking autophagy impairment to the central nervous system, for example, senescence and diseases (acute, chronic, and autoimmunity-mediated neurodegeneration, including Alzheimer’s, Parkinson’s, and Huntington’s diseases, and multiple sclerosis).

Proliferation and activation of microglia in the brain, mostly around amyloid plaques, is a prominent feature of Alzheimer’s disease [132]. Human genetic data point to a key role of microglia in the pathogenesis of Alzheimer’s disease. Microglia have been suggested to be a major contributor to neural development, neuroinflammation, and degeneration. Dysregulated immunoactivity in Alzheimer’s disease has been broadly studied, and the latest research on animal models has identified a new cluster of microglia (disease-associated microglia) alongside previously detected glial populations (e.g., plaque-associated microglia and dark microglia) [133].

Alzheimer’s disease is associated with microglia-mediated neuroinflammation and with macrophagic activity. Amyloid β (Aβ) is a peptide fragment of amyloid precursor protein and triggers the progression of Alzheimer’s disease. According to one hypothesis, Aβ can contribute to neurodegeneration in several ways, including mitochondrial dysfunction, oxidative stress, and brain insulin resistance. Therefore, protecting neurons from Aβ-induced neurotoxicity is an effective strategy for attenuating Alzheimer’s disease pathogenesis, as demonstrated recently by means of a novel intervention reducing Aβ neurotoxicity in the brain. Recent studies, revealing transcriptomic clusters of disease-related cells and involving an analysis of sequenced RNA from sorted myeloid cells, seem to support the hypothesis of the crucial involvement of glia in the pathogenesis of Alzheimer’s disease [132].

Parkinson’s disease is the most common neurodegenerative movement disorder, which is characterized neuropathologically by the loss of dopaminergic neurons in the substantia nigra pars compacta and by the presence of Lewy bodies containing α-synuclein [134]. Using a model of Parkinson’s disease based on transgenic overexpression of α-synuclein, it was demonstrated that accumulation of A53T-mutant α-synuclein induces three autophagy cell responses; namely, the inhibition of autophagy caused by the accumulation of α-synuclein, compensatory activation of macroautophagy in response to inhibition of chaperone-mediated autophagy, and toxic effects of mutant α-synuclein accompanied by the activation of autophagy. Low autophagic activity was demonstrated in the dopaminergic structures of 5-month-old transgenic B6 Cg-Tg(Prnp-SNCA*A53T)23Mkle/J mice as compared to control C57Bl/6J mice [135].

The neuroprotective effect of autophagy activation by rapamycin and TH has also been demonstrated in a mouse model of Parkinson’s disease induced by neurotoxin 1-methyl-4-phenyl-1,2,3,6-tetrahydropyridine (MPTP). Both rapamycin (10 mg/kg per day, 7 days) and TH (2% in drinking water, 7 days) increased the expression of LC3-II (a marker of autophagy activation) in the frontal cortex and striatum of normal C57Bl/6J mice [136]. It was concluded that autophagy activation by the combination of rapamycin and TH through different pathways reversed both neuronal dopaminergic and behavioral deficits in vivo and that this approach may be a promising therapy for Parkinson’s disease-like pathology. The therapeutic effect of TH on neurodegeneration was discussed in terms of whether TH is an inducer or inhibitor of autophagy (i.e., disturbing the fusion of autophagolysosomes and autolysosomes; known as ‘lysosomal flux’) [126].

## 4. Conclusions

In this review, new data have been presented on several drugs used in medicine that exhibit lysosomotropic features. Data obtained by us from in vivo experiments with different LA demonstrated harmful side effects, which included lysosomal overloading and increased permeability of lysosomal membranes during intralysosomal storage syndrome, which was followed by changes in cell metabolism. Experimental animal models using LA drugs often resemble lysosomal storage diseases and the associated consequences of storage syndrome treatment, especially in the case of tolerance to treatment by enzyme replacement. Lysosomotropic pharmaceutical drugs that are basic and lipophilic typically become sequestered inside lysosomes.

Some authors in this review have considered the effects of LA in vivo as lysosomal adaptation to stress, which has not been well-characterized to date. Lysosomotropic drugs have also induced lysosomal dysfunction, which is exemplified by increased cathepsin activity, increased LAMP2 staining, and intracellular and extracellular substrate accumulation, including phospholipids, SQSTM1/p62, and glyceraldehyde 3-phosphate dehydrogenase (GAPDH). Lysosomal activation can be attributed to lysosomal dysfunction, leading to compensatory responses with nuclear translocation of transcriptional factors (such as TFEB and others). Potentially, LA compounds can evoke a compensatory lysosomal biogenic response with the ultimate consequence being lysosomal functional impairment.

The common feature of many LA is the development of autophagy, which was described in this review. Specific sections of this review were devoted to ‘inducers’ of autophagy, which represents a new direction in cell biology that is focused on intentionally exploiting autophagy activation for therapeutic reasons. Stimulation of lipophagy with the assistance of inducers may potentially be useful in the treatment of diseases characterized by intralysosomal storage of lipids, such as liver steatosis, atherosclerosis, and some lysosomal storage diseases.

This review also provided information regarding lysosomotropic metals. Gadolinium compounds (widely used in magnetic resonance imaging) accumulate in lysosomes and remain for an extended period following administration, although most of the gadolinium is eliminated by 24 h post dosing. In various experiments, it has been shown that low concentrations of gadolinium can be detected in liver cells even 30 days after a single administration to mice. These data suggest that other lysosomotropic metals (such as plutonium) would accumulate in lysosomes and may potentially remain in the body for an extended period.

Intralysosomal storage of some metals (e.g., cadmium) from industrial waste, even in low concentration, is dangerous. Interestingly, other LA (e.g., gadolinium-based compounds) protect KC (possibly via macrophage depression) from cadmium toxicity. The mechanism(s) underlying this protection certainly requires further investigation.

## Figures and Tables

**Table 1:** Medical drugs: lysosomotropic agents and modulators of autophagy.

Name and Basic Functions of the Drug or Compound	Biological Effects on Lysosomes	References
Chloroquine, antimalarial, antiparasitic, anti-inflammatory effects; antiviral activities in vitro against viruses, including coronaviruses, dengue virus and the biosafety level 4 *Nipah* and *Hendra* paramyxoviruses. The in vivo efficacy in the treatment of COVID-19 is currently a matter of debate.	Intralysosomal pH alteration; lysosomal membrane permeabilization; inhibitor of autophagic flux. Inhibition of acidic proteases involved in the maturation of virus fusion protein.	Schneider et al., 1997 [5]; Pisonero-Vaquero, Medina, 2017 [2] Carrière et al., 2020 [6] Blaess et al., 2020 [7] Norinder et al., 2020 [8]
Hydroxychloroquine, antiviral effect in vitro, similar to chloroquine. The in vivo efficacy of hydroxychloroquine in the treatment of COVID-19 is currently a matter of debate.	Similar to chloroquine (but no phospholipidosis).	Carrière et al., 2020 [6] Blaess et al., 2020 [7] Norinder et al., 2020 [8]
Triton WR 1339 (Tyloxapol), non-ionic surfactant, additive in pharmacy; PEGylated Tyloxapol niosomes for the controlled release of first-line anti-tuberculosis drugs.	Increased autophagy (induced by macrocyclon); lysosomal membrane permeabilization, lysosomal swelling	Plattner et al., 1975 [9] Schneider et al., 1997 [5]
Suramin (antiparasitic drug, to treat African sleeping sickness or trypanosomiasis. It works by inhibiting ATP signaling).	Accumulation in lysosomes; inhibitor of lysosomal enzymes, suppressing phagosome-lysosome fusion; is commonly employed as a tool for inducing experimental mucopolysaccharidosis and lipidosis.	Panagiotidis et al., 1991 [10] Schneider et al., 1997 [5] Constantopoulos et al., 1980 [11] Korolenko et al., 1981 [12]
Metformin (anti-aging; anti-diabetic; anti-tumor).	Prevents cell tumor through autophagy (by AMPK activation and inhibition of mTOR, major inhibitor of autophagic flux); able to block the autophagosome fusion with lysosomes.	De Santi et al., 2019 [13]
Gold sodium thiomalate, anti-inflammatory effect in rheumatoid arthritis, active treatment in severe cases (in children).	Effect on lysosomal enzyme activity in macrophages and lysosomal membrane, bacterial killing.	Schneider et al., 1997 [5]
PVP, for blood transfusion; (polyvinyl pyrrolidone)-coated AgNPs have an anti-leukemia effect against human myeloid leukemia cells.	Accumulation inside of lysosomes, lysosomal membrane stability changes; trigger of autophagy.	Soares et al., 2016 [14]
Resveratrol.	Enhancer of autophagy in human U373 glioma cells.	Yamamoto et al., 2010 [15]
Trehalose.	Disaccharide trehalose is a novel inducer of TFEB with atheroprotective effects. mTOR (the mammalian target of rapamycin)-independent inducer of autophagy. Potent blocker of the autophagic flux.	Evans et al., 2018 [16] Belzile et al., 2016 [17] Yoon et al., 2017 [18]
Imipramine (antidepressant), cationic amphiphilic drug.	Inhibition of lysosomal ceramide-producing enzyme, acid sphingomyelinase with imipramine or by various stresses are involved in cell death and has been implicated in autophagy.	Justice et al., 2018 [19]
Amitriptyline (antidepressant) Amiodarone (anti-arrhythmic), Strong cationic amphiphilic drug.	Autophagy inducer (neurons). Amiodarone inhibits autophagic flux and prevents tube formation in vascular endothelial cells. Amiodarone increased autophagic flux and apoptosis in H460 human lung epithelial cells and BEAS-2B normal human bronchial epithelial cells.	Gulbins et al., 2018 [20] Guan et al., 2019 [21] Lee et al., 2013 [22] Easwaranathan et al., 2019 [23]
Azithromycin (antibiotic) Strong cationic amphiphilic drug.	Excessive lysosomal ion-trapping. In chronic administration in vivo pharmacological blockade of autophagy.	Halliwell, 1997 [24] Derendorf, 2020 [25] Salata et al., 2013 [26] Renna et al., 2011 [27] Mukai et al., 2016 [28]
Erythromycin (antibiotic) Moderate cationic drug.	Accumulation inside of cells (in lysosomes of phagocytic cells).	Carlier et al., 1987 [29]
Rapamycin.	Induces autophagy in human neuroblastoma cells.	Lin et al. 2018 [30]

## References

[1] de Duve C., de Barsy T., Poole B., Trouet A., Tulkens P., Van Hoof F. (1974). Commentary. Lysosomotropic agents. Biochem. Pharmacol..

[2] Pisonero-Vaquero S., Medina D.L. (2017). Lysosomotropic drugs: Pharmacological tools to study lysosomal function. Curr. Drug Metab..

[3] Pourahmad J., Hosseini M.J., Eskandari M.R., Rahmani F. (2012). Involvement of four different intracellular sites in chloroacetaldehyde- induced oxidative stress cytotoxicity. Iran. J. Pharm. Res..

[4] Kuzu O.F., Toprak M., Noory M.A., Robertson G.P. (2017). Effect of lysosomotropic molecules on cellular homeostasis. Pharmacol. Res..

[5] Schneider P., Korolenko T.A., Busch U. (1997). A review of drug-induced lysosomal disorders of the liver in man and laboratory animals. Microsc. Res. Tech..

[6] Carriere F., Longhi S., Record M. (2020). The endosomal lipid bis(monoacylglycero) phosphate as a potential key player in the mechanism of action of chloroquine against SARS-COV-2 and other enveloped viruses hijacking the endocytic pathway. Biochimie.

[7] Blaess M., Kaiser L., Sauer M., Csuk R., Deigner H.P. (2020). COVID-19/SARS-CoV-2 Infection: Lysosomes and Lysosomotropism Implicate New Treatment Strategies and Personal Risks. Int. J. Mol. Sci..

[8] Norinder U., Tuck A., Norgren K., Munic Kos V. (2020). Existing highly accumulating lysosomotropic drugs with potential for repurposing to target COVID-19. Biomed. Pharmacother..

[9] Plattner H., Henning R., Brauser B. (1975). Formation of triton WR 1339-filled rat liver lysosomes. II. Involvement of autophagy and of pre-existing lysosomes. Exp. Cell Res..

[10] Panagiotidis G., Salehi A.A., Lundquist I. (1991). Effect of the lysosomotropic drug suramin on islet lysosomal enzyme activities and the insulin-secretory response induced by various secretagogues. Pharmacology.

[11] Constantopoulos G., Rees S., Cragg B.G., Barranger J.A., Brady R.O. (1980). Experimental animal model for mucopolysaccharidosis: Suramin-induced glycosaminoglycan and sphingolipid accumulation in the rat. Proc. Natl. Acad. Sci. USA.

[12] Korolenko T.A., Pupyshev A.B., Malygin A.E. (1981). Heterophagic function and rate of intralysosomal proteolysis during lysosomotropic agents administration. Acta Biol. Med. Ger..

[13] De Santi M., Baldelli G., Diotallevi A., Galluzzi L., Schiavano G.F., Brandi G. (2019). Metformin prevents cell tumorigenesis through autophagy-related cell death. Sci. Rep..

[14] Soares T., Ribeiro D., Proenca C., Chiste R.C., Fernandes E., Freitas M. (2016). Size-dependent cytotoxicity of silver nanoparticles in human neutrophils assessed by multiple analytical approaches. Life Sci..

[15] Yamamoto M., Suzuki S.O., Himeno M. (2010). Resveratrol-induced autophagy in human U373 glioma cells. Oncol. Lett..

[16] Evans T.D., Jeong S.J., Zhang X., Sergin I., Razani B. (2018). TFEB and trehalose drive the macrophage autophagy-lysosome system to protect against atherosclerosis. Autophagy.

[17] Belzile J.P., Sabalza M., Craig M., Clark E., Morello C.S., Spector D.H. (2016). Trehalose, an mTOR-independent inducer of autophagy, inhibits human cytomegalovirus infection in multiple cell types. J. Virol..

[18] Yoon Y.S., Cho E.D., Jung Ahn W., Won Lee K., Lee S.J., Lee H.J. (2017). Is trehalose an autophagic inducer? Unraveling the roles of non-reducing disaccharides on autophagic flux and alpha-synuclein aggregation. Cell Death Dis..

[19] Justice M.J., Bronova I., Schweitzer K.S., Poirier C., Blum J.S., Berdyshev E.V., Petrache I. (2018). Inhibition of acid sphingomyelinase disrupts LYNUS signaling and triggers autophagy. J. Lipid Res..

[20] Gulbins A., Schumacher F., Becker K.A., Wilker B., Soddemann M., Boldrin F., Muller C.P., Edwards M.J., Goodman M., Caldwell C.C. (2018). Antidepressants act by inducing autophagy controlled by sphingomyelin-ceramide. Mol. Psychiatry.

[21] Guan Y., Li X., Umetani M., Boini K.M., Li P.L., Zhang Y. (2019). Tricyclic antidepressant amitriptyline inhibits autophagic flux and prevents tube formation in vascular endothelial cells. Basic Clin. Pharmacol. Toxicol..

[22] Lee K.Y., Oh S., Choi Y.J., Oh S.H., Yang Y.S., Yang M.J., Lee K., Lee B.H. (2013). Activation of autophagy rescues amiodarone-induced apoptosis of lung epithelial cells and pulmonary toxicity in rats. Toxicol. Sci..

[23] Easwaranathan A., Inci B., Ulrich S., Brunken L., Nikiforova V., Norinder U., Swanson S., Munic Kos V. (2019). Quantification of Intracellular Accumulation and Retention of Lysosomotropic Macrocyclic Compounds by High-Throughput Imaging of Lysosomal Changes. J. Pharm. Sci..

[24] Halliwell W.H. (1997). Cationic amphiphilic drug-induced phospholipidosis. Toxicol. Pathol..

[25] Derendorf H. (2020). Excessive lysosomal ion-trapping of hydroxychloroquine and azithromycin. Int. J. Antimicrob. Agents.

[26] Salata C., Calistri A., Parolin C., Baritussio A., Palu G. (2017). Antiviral activity of cationic amphiphilic drugs. Expert Rev. Anti Infect. Ther..

[27] Renna M., Schaffner C., Brown K., Shang S., Tamayo M.H., Hegyi K., Grimsey N.J., Cusens D., Coulter S., Cooper J. (2011). Azithromycin blocks autophagy and may predispose cystic fibrosis patients to mycobacterial infection. J. Clin. Investig..

[28] Mukai S., Moriya S., Hiramoto M., Kazama H., Kokuba H., Che X.F., Yokoyama T., Sakamoto S., Sugawara A., Sunazuka T. (2016). Macrolides sensitize EGFR-TKI-induced non-apoptotic cell death via blocking autophagy flux in pancreatic cancer cell lines. Int. J. Oncol..

[29] Carlier M.B., Zenebergh A., Tulkens P.M. (1987). Cellular uptake and subcellular distribution of roxithromycin and erythromycin in phagocytic cells. J. Antimicrob. Chemother..

[30] Lin X., Han L., Weng J., Wang K., Chen T. (2018). Rapamycin inhibits proliferation and induces autophagy in human neuroblastoma cells. Biosci. Rep..

[31] Ward C., Martinez-Lopez N., Otten E.G., Carroll B., Maetzel D., Singh R., Sarkar S., Korolchuk V.I. (2016). Autophagy, lipophagy and lysosomal lipid storage disorders. Biochim. Biophys. Acta.

[32] Galluzzi L., Bravo-San Pedro J.M., Levine B., Green D.R., Kroemer G. (2017). Pharmacological modulation of autophagy: Therapeutic potential and persisting obstacles. Nat. Rev. Drug Discov..

[33] Sergin I., Evans T.D., Zhang X., Bhattacharya S., Stokes C.J., Song E., Ali S., Dehestani B., Holloway K.B., Micevych P.S. (2017). Exploiting macrophage autophagy-lysosomal biogenesis as a therapy for atherosclerosis. Nat. Commun..

[34] Qiu P., Liu Y., Zhang J. (2019). Review: The role and mechanisms of macrophage autophagy in sepsis. Inflammation.

[35] Seebacher F., Zeigerer A., Kory N., Krahmer N. (2020). Hepatic lipid droplet homeostasis and fatty liver disease. Semin. Cell Dev. Biol..

[36] Yang N., Shen H.M. (2020). Targeting the endocytic pathway and autophagy process as a novel therapeutic strategy in COVID-19. Int. J. Biol. Sci..

[37] Kumar M., Ojha S., Rai P., Joshi A., Kamat S.S., Mallik R. (2019). Insulin activates intracellular transport of lipid droplets to release triglycerides from the liver. J. Cell Biol..

[38] Gluchowski N.L., Becuwe M., Walther T.C., Farese R.V. (2017). Lipid droplets and liver disease: From basic biology to clinical implications. Nat. Rev. Gastroenterol. Hepatol..

[39] Li Y., Liu R., Wu J., Li X. (2020). Self-eating: Friend or foe? The emerging role of autophagy in fibrotic diseases. Theranostics.

[40] Escamilla-Ramirez A., Castillo-Rodriguez R.A., Zavala-Vega S., Jimenez-Farfan D., Anaya-Rubio I., Briseno E., Palencia G., Guevara P., Cruz-Salgado A., Sotelo J. (2020). Autophagy as a potential therapy for malignant glioma. Pharmaceuticals.

[41] Liu K., Czaja M.J. (2013). Regulation of lipid stores and metabolism by lipophagy. Cell Death Differ..

[42] Singh R., Cuervo A.M. (2012). Lipophagy: Connecting autophagy and lipid metabolism. Int. J. Cell Biol..

[43] Ouimet M., Franklin V., Mak E., Liao X., Tabas I., Marcel Y.L. (2011). Autophagy regulates cholesterol efflux from macrophage foam cells via lysosomal acid lipase. Cell Metab..

[44] Jeong S.J., Lee M.N., Oh G.T. (2017). The role of macrophage lipophagy in reverse cholesterol transport. Endocrinol. Metab..

[45] Kounakis K., Chaniotakis M., Markaki M., Tavernarakis N. (2019). Emerging roles of lipophagy in health and disease. Front. Cell Dev. Biol..

[46] Levine B., Kroemer G. (2008). Autophagy in the pathogenesis of disease. Cell.

[47] Kim Y.S., Silwal P., Kim S.Y., Yoshimori T., Jo E.K. (2019). Autophagy-activating strategies to promote innate defense against mycobacteria. Exp. Mol. Med..

[48] Levine B., Packer M., Codogno P. (2015). Development of autophagy inducers in clinical medicine. J. Clin. Investig..

[49] Stark M., Silva T.F.D., Levin G., Machuqueiro M., Assaraf Y.G. (2020). The lysosomotropic activity of hydrophobic weak base drugs is mediated via their intercalation into the lysosomal membrane. Cells.

[50] Melo F.R., Lundequist A., Calounova G., Wernersson S., Pejler G. (2011). Lysosomal membrane permeabilization induces cell death in human mast cells. Scand. J. Immunol..

[51] Villamil Giraldo A.M., Appelqvist H., Ederth T., Ollinger K. (2014). Lysosomotropic agents: Impact on lysosomal membrane permeabilization and cell death. Biochem. Soc. Trans..

[52] Mauthe M., Orhon I., Rocchi C., Zhou X., Luhr M., Hijlkema K.J., Coppes R.P., Engedal N., Mari M., Reggiori F. (2018). Chloroquine inhibits autophagic flux by decreasing autophagosome-lysosome fusion. Autophagy.

[53] Korolenko T.A., Rukavishnikova E.V., Safina A.F., Dushkin M.I., Mynkina G.I. (1992). Endocytosis by liver cells during suppression of intralysosomal proteolysis. Biol. Chem. Hoppe Seyler.

[54] Raley M.J., Schwacha M.G., Loegering D.J. (1997). Lysosomotropic agents ameliorate macrophage dysfunction following the phagocytosis of IgG-coated erythrocytes: A role for lipid peroxidation. Inflammation.

[55] Dielschneider R.F., Henson E.S., Gibson S.B. (2017). Lysosomes as oxidative targets for cancer therapy. Oxid. Med. Cell Longev..

[56] Parks A., Marceau F. (2016). Lysosomotropic cationic drugs induce cytostatic and cytotoxic effects: Role of liposolubility and autophagic flux and antagonism by cholesterol ablation. Toxicol. Appl. Pharmacol..

[57] Zhitomirsky B., Yunaev A., Kreiserman R., Kaplan A., Stark M., Assaraf Y.G. (2018). Lysosomotropic drugs activate TFEB via lysosomal membrane fluidization and consequent inhibition of mTORC1 activity. Cell Death Dis..

[58] Giuliano S., Dufies M., Ndiaye P.D., Viotti J., Borchiellini D., Parola J., Vial V., Cormerais Y., Ohanna M., Imbert V. (2019). Resistance to lysosomotropic drugs used to treat kidney and breast cancers involves autophagy and inflammation and converges in inducing CXCL5. Theranostics.

[59] Papanagnou P., Papadopoulos G.E., Stivarou T., Pappas A. (2019). Toward fully exploiting the therapeutic potential of marketed pharmaceuticals: The use of octreotide and chloroquine in oncology. Onco Targets Ther..

[60] Lubow C., Bockstiegel J., Weindl G. (2020). Lysosomotropic drugs enhance pro-inflammatory responses to IL-1beta in macrophages by inhibiting internalization of the IL-1 receptor. Biochem. Pharmacol..

[61] Lamoureux F., Thomas C., Crafter C., Kumano M., Zhang F., Davies B.R., Gleave M.E., Zoubeidi A. (2013). Blocked autophagy using lysosomotropic agents sensitizes resistant prostate tumor cells to the novel Akt inhibitor AZD5363. Clin. Cancer Res..

[62] Sharma N., Thomas S., Golden E.B., Hofman F.M., Chen T.C., Petasis N.A., Schonthal A.H., Louie S.G. (2012). Inhibition of autophagy and induction of breast cancer cell death by mefloquine, an antimalarial agent. Cancer Lett..

[63] Jacques P.J. (1976). The selection and design of lysosomotropic drugs. Adv. Exp. Med. Biol..

[64] Jacques P.J., Lambert F. (1979). Alterations of rat liver lysosomes after treatment with particulate beta 1-3 glucan from Saccharomyces cerevisiae. Adv. Exp. Med. Biol..

[65] Wu M.Y., Lu J.H. (2020). Autophagy and macrophage functions: Inflammatory response and phagocytosis. Cells.

[66] Schulze R.J., Sathyanarayan A., Mashek D.G. (2017). Breaking fat: The regulation and mechanisms of lipophagy. Biochim. Biophys. Acta Mol. Cell Biol. Lipids.

[67] Hazari Y., Bravo-San Pedro J.M., Hetz C., Galluzzi L., Kroemer G. (2020). Autophagy in hepatic adaptation to stress. J. Hepatol..

[68] Umahara T., Uchihara T., Hirao K., Shimizu S., Hashimoto T., Kohno M., Hanyu H. (2020). Essential autophagic protein Beclin 1 localizes to atherosclerotic lesions of human carotid and major intracranial arteries. J. Neurol. Sci..

[69] Levine B., Kroemer G. (2019). Biological functions of autophagy genes: A disease perspective. Cell.

[70] Parzych K.R., Klionsky D.J. (2014). An overview of autophagy: Morphology, mechanism, and regulation. Antioxid. Redox Signal..

[71] Saha S., Panigrahi D.P., Patil S., Bhutia S.K. (2018). Autophagy in health and disease: A comprehensive review. Biomed. Pharmacother..

[72] Fernández Á.F., López-Otín C. (2015). The functional and pathologic relevance of autophagy proteases. J. Clin. Investig..

[73] Weiss-Sadan T., Maimoun D., Oelschlagel D., Kaschani F., Misiak D., Gaikwad H., Ben-Nun Y., Merquiol E., Anaki A., Tsvirkun D. (2019). Cathepsins drive anti-inflammatory activity by regulating autophagy and mitochondrial dynamics in macrophage foam cells. Cell Physiol. Biochem..

[74] Ballabio A., Bonifacino J.S. (2020). Lysosomes as dynamic regulators of cell and organismal homeostasis. Nat. Rev. Mol. Cell Biol..

[75] Cui W., Sathyanarayan A., Lopresti M., Aghajan M., Chen C., Mashek D.G. (2020). Lipophagy-derived fatty acids undergo extracellular efflux via lysosomal exocytosis. Autophagy.

[76] Zhang Z., Yao Z., Chen Y., Qian L., Jiang S., Zhou J., Shao J., Chen A., Zhang F., Zheng S. (2018). Lipophagy and liver disease: New perspectives to better understanding and therapy. Biomed. Pharmacother..

[77] Martinez-Lopez N., Singh R. (2015). Autophagy and lipid droplets in the liver. Annu. Rev. Nutr..

[78] Shi L., Wang K., Deng Y., Wang Y., Zhu S., Yang X., Liao W. (2019). Role of lipophagy in the regulation of lipid metabolism and the molecular mechanism. Nan Fang Yi Ke Da Xue Xue Bao.

[79] Goeritzer M., Vujic N., Schlager S., Chandak P.G., Korbelius M., Gottschalk B., Leopold C., Obrowsky S., Rainer S., Doddapattar P. (2015). Active autophagy but not lipophagy in macrophages with defective lipolysis. Biochim. Biophys. Acta.

[80] Carr R.M., Ahima R.S. (2016). Pathophysiology of lipid droplet proteins in liver diseases. Exp. Cell Res..

[81] Mishra S., Khaddaj R., Cottier S., Stradalova V., Jacob C., Schneiter R. (2016). Mature lipid droplets are accessible to ER luminal proteins. J. Cell Sci..

[82] Elrick M.J., Lieberman A.P. (2013). Autophagic dysfunction in a lysosomal storage disorder due to impaired proteolysis. Autophagy.

[83] Vanier M.T. (2010). Niemann-Pick disease type C. Orphanet. J. Rare Dis..

[84] Sleat D.E., Wiseman J.A., Sohar I., El-Banna M., Zheng H., Moore D.F., Lobel P. (2012). Proteomic analysis of mouse models of Niemann-Pick C disease reveals alterations in the steady-state levels of lysosomal proteins within the brain. Proteomics.

[85] Cluzeau C.V., Watkins-Chow D.E., Fu R., Borate B., Yanjanin N., Dail M.K., Davidson C.D., Walkley S.U., Ory D.S., Wassif C.A. (2012). Microarray expression analysis and identification of serum biomarkers for Niemann-Pick disease, type C1. Hum. Mol. Genet..

[86] Meyers A., Weiskittel T.M., Dalhaimer P. (2017). Lipid droplets: Formation to breakdown. Lipids.

[87] Jakos T., Pislar A., Pecar Fonovic U., Kos J. (2020). Lysosomal peptidases in innate immune cells: Implications for cancer immunity. Cancer Immunol. Immunother..

[88] Chen N., Ou Z., Zhang W., Zhu X., Li P., Gong J. (2018). Cathepsin B regulates non-canonical NLRP3 inflammasome pathway by modulating activation of caspase-11 in Kupffer cells. Cell Prolif..

[89] Sleyster E.C., Knook D.L. (1982). Relation between localization and function of rat liver Kupffer cells. Lab. Investig..

[90] Ferland G., Perea A., Audet M., Tuchweber B. (1990). Characterization of liver lysosomal enzyme activity in hepatocytes, Kupffer and endothelial cells during aging: Effect of dietary restriction. Mech. Ageing Dev..

[91] Ansorge S., Wiederanders B., Riemann S., Brouwer A., Knook D.L. (1984). Distribution of thiol-protein disulfide oxidoreductase, insulin-glucagon proteinase and cathepsin D in different cell types of the rat liver. Biomed. Biochim. Acta.

[92] Shiozaki M., Hayakawa N., Shibata M., Koike M., Uchiyama Y., Gotow T. (2011). Closer association of mitochondria with lipid droplets in hepatocytes and activation of Kupffer cells in resveratrol-treated senescence-accelerated mice. Histochem. Cell Biol..

[93] Hardonk M.J., Dijkhuis F.W., Hulstaert C.E., Koudstaal J. (1992). Heterogeneity of rat liver and spleen macrophages in gadolinium chloride-induced elimination and repopulation. J. Leukoc. Biol..

[94] Zhu R., Guo W., Fang H., Cao S., Yan B., Chen S., Zhang K., Zhang S. (2018). Kupffer cell depletion by gadolinium chloride aggravates liver injury after brain death in rats. Mol. Med. Rep..

[95] Akai S., Uematsu Y., Tsuneyama K., Oda S., Yokoi T. (2016). Kupffer cell-mediated exacerbation of methimazole-induced acute liver injury in rats. J. Appl. Toxicol..

[96] Liu C., Yang Z., Wang L., Lu Y., Tang B., Miao H., Xu Q., Chen X. (2015). Combination of sorafenib and gadolinium chloride (GdCl3) attenuates dimethylnitrosamine(DMN)-induced liver fibrosis in rats. BMC Gastroenterol..

[97] Song M., Schuschke D.A., Zhou Z., Zhong W., Zhang J., Zhang X., Wang Y., McClain C.J. (2015). Kupffer cell depletion protects against the steatosis, but not the liver damage, induced by marginal-copper, high-fructose diet in male rats. Am. J. Physiol. Gastrointest. Liver Physiol..

[98] Kiris I., Okutan H., Savas C., Yonden Z., Delibas N. (2007). Gadolinium chloride attenuates aortic occlusion-reperfusion-induced myocardial injury in rats. Saudi Med. J..

[99] Zhu F., Li X., Jiang Y., Zhu H., Zhang H., Zhang C., Zhao Y., Luo F. (2015). GdCl3 suppresses the malignant potential of hepatocellular carcinoma by inhibiting the expression of CD206 in tumorassociated macrophages. Oncol. Rep..

[100] Gordon S., Martinez F.O. (2010). Alternative activation of macrophages: Mechanism and functions. Immunity.

[101] Singh R., Kaushik S., Wang Y., Xiang Y., Novak I., Komatsu M., Tanaka K., Cuervo A.M., Czaja M.J. (2009). Autophagy regulates lipid metabolism. Nature.

[102] Settembre C., Ballabio A. (2014). Lysosome: Regulator of lipid degradation pathways. Trends Cell Biol..

[103] Johnston T.P., Korolenko T.A., Sahebkar A. (2017). P-407-induced mouse model of dose-controlled hyperlipidemia and atherosclerosis: 25 years later. J. Cardiovasc. Pharmacol..

[104] Winchester B. (2014). Lysosomal diseases: Diagnostic update. J. Inherit. Metab. Dis..

[105] Winchester B., Vellodi A., Young E. (2000). The molecular basis of lysosomal storage diseases and their treatment. Biochem. Soc. Trans..

[106] Norinder U., Munic Kos V. (2019). QSAR models for predicting five levels of cellular accumulation of lysosomotropic macrocycles. Int. J. Mol. Sci..

[107] Leighton F., Poole B., Beaufay H., Baudhuin P., Coffey J.W., Fowler S., De Duve C. (1968). The large-scale separation of peroxisomes, mitochondria, and lysosomes from the livers of rats injected with triton WR-1339. Improved isolation procedures, automated analysis, biochemical and morphological properties of fractions. J. Cell Biol..

[108] Wattiaux R., Wibo M., Baudhuin P., de Reuck M.Sc A.V.S., Margrate P., Cameron M.A. (1963). Influence of the injection of Triton WR-1339 on the properties of rat liver lysosomes. Ciba Foundation Symposium: Lysosomes.

[109] Hayashi H., Niinobe S., Matsumoto Y., Suga T. (1981). Effects of Triton WR-1339 on lipoprotein lipolytic activity and lipid content of rat liver lysosomes. J. Biochem..

[110] Millar J.S., Cromley D.A., McCoy M.G., Rader D.J., Billheimer J.T. (2005). Determining hepatic triglyceride production in mice: Comparison of poloxamer 407 with Triton WR-1339. J. Lipid Res..

[111] Lu S., Sung T., Lin N., Abraham R.T., Jessen B.A. (2017). Lysosomal adaptation: How cells respond to lysosomotropic compounds. PLoS ONE.

[112] Abdou H.M., Yousef M.I., Newairy A.A. (2018). Triton WR-1339-induced hyperlipidemia, DNA fragmentation, neurotransmitters inhibition, oxidative damage, histopathological and morphometric changes: The protective role of soybean oil. J. Basic Appl. Zool..

[113] Korolenko T.A., Johnston T.P., Machova E., Bgatova N.P., Lykov A.P., Goncharova N.V., Nescakova Z., Shintyapina A.B., Maiborodin I.V., Karmatskikh O.L. (2018). Hypolipidemic effect of mannans from C. albicans serotypes a and B in acute hyperlipidemia in mice. Int. J. Biol. Macromol..

[114] Serrano-Puebla A., Boya P. (2018). Lysosomal membrane permeabilization as a cell death mechanism in cancer cells. Biochem. Soc. Trans..

[115] Kuo J.H., Jan M.S., Chiu H.W. (2006). Cytotoxic properties of tyloxapol. Pharm. Res..

[116] Huterer S., Phillips M.J., Wherrett J.R. (1975). Effects of prolonged administration of triton WR-1339 to the rat on morphology and phospholipids of liver. Lab. Investig..

[117] Russo M., Russo G.L. (2018). Autophagy inducers in cancer. Biochem. Pharmacol..

[118] Shoji-Kawata S., Sumpter R., Leveno M., Campbell G.R., Zou Z., Kinch L., Wilkins A.D., Sun Q., Pallauf K., MacDuff D. (2013). Identification of a candidate therapeutic autophagy-inducing peptide. Nature.

[119] Remmerie A., Martens L., Scott C.L. (2020). Macrophage subsets in obesity, aligning the liver and adipose tissue. Front. Endocrinol..

[120] Marschallinger J., Iram T., Zardeneta M., Lee S.E., Lehallier B., Haney M.S., Pluvinage J.V., Mathur V., Hahn O., Morgens D.W. (2020). Lipid-droplet-accumulating microglia represent a dysfunctional and proinflammatory state in the aging brain. Nat. Neurosci..

[121] Lee G.C., Lin C.H., Tao Y.C., Yang J.M., Hsu K.C., Huang Y.J., Huang S.H., Kung P.J., Chen W.L., Wang C.M. (2015). The potential of lactulose and melibiose, two novel trehalase-indigestible and autophagy-inducing disaccharides, for polyQ-mediated neurodegenerative disease treatment. Neurotoxicology.

[122] Fan Y., Wang N., Rocchi A., Zhang W., Vassar R., Zhou Y., He C. (2017). Identification of natural products with neuronal and metabolic benefits through autophagy induction. Autophagy.

[123] Chiarelli R., Roccheri M.C. (2012). Heavy metals and metalloids as autophagy inducing agents: Focus on cadmium and arsenic. Cells.

[124] Takanezawa Y., Nakamura R., Kusaka T., Ohshiro Y., Uraguchi S., Kiyono M. (2020). Significant contribution of autophagy in mitigating cytotoxicity of gadolinium ions. Biochem. Biophys. Res. Commun..

[125] la Fuente F.P., Quezada L., Sepulveda C., Monsalves-Alvarez M., Rodriguez J.M., Sacristan C., Chiong M., Llanos M., Espinosa A., Troncoso R. (2019). Exercise regulates lipid droplet dynamics in normal and fatty liver. Biochim. Biophys. Acta Mol. Cell Biol. Lipids.

[126] Lee H.J., Yoon Y.S., Lee S.J. (2018). Mechanism of neuroprotection by trehalose: Controversy surrounding autophagy induction. Cell Death Dis..

[127] Seranova E., Connolly K.J., Zatyka M., Rosenstock T.R., Barrett T., Tuxworth R.I., Sarkar S. (2017). Dysregulation of autophagy as a common mechanism in lysosomal storage diseases. Essays Biochem..

[128] Glick D., Barth S., Macleod K.F. (2010). Autophagy: Cellular and molecular mechanisms. J. Pathol..

[129] Plaza-Zabala A., Sierra-Torre V., Sierra A. (2017). Autophagy and microglia: Novel partners in neurodegeneration and aging. Int. J. Mol. Sci..

[130] Mo Y., Sun Y.Y., Liu K.Y. (2020). Autophagy and inflammation in ischemic stroke. Neural Regen. Res..

[131] Gopinath A., Collins A., Khoshbouei H., Streit W. (2020). Microglia and other myeloid cells in CNS health and disease. J. Pharmacol. Exp. Ther..

[132] Hansen D.V., Hanson J.E., Sheng M. (2018). Microglia in Alzheimer’s disease. J. Cell Biol..

[133] Hashemiaghdam A., Mroczek M. (2020). Microglia heterogeneity and neurodegeneration: The emerging paradigm of the role of immunity in Alzheimer’s disease. J. Neuroimmunol..

[134] Giaime E., Tong Y., Wagner L.K., Yuan Y., Huang G., Shen J. (2017). Age-dependent dopaminergic neurodegeneration and impairment of the autophagy-lysosomal pathway in LRRK-deficient mice. Neuron.

[135] Pupyshev A.B., Korolenko T.A., Akopyan A.A., Amstislavskaya T.G., Tikhonova M.A. (2018). Suppression of autophagy in the brain of transgenic mice with overexpression of capital A, Cyrillic53capital TE, Cyrillic-mutant alpha-synuclein as an early event at synucleinopathy progression. Neurosci. Lett..

[136] Pupyshev A.B., Tikhonova M.A., Akopyan A.A., Tenditnik M.V., Dubrovina N.I., Korolenko T.A. (2019). Therapeutic activation of autophagy by combined treatment with rapamycin and trehalose in a mouse MPTP-induced model of Parkinson’s disease. Pharmacol. Biochem. Behav..

